# Multimodal Prehabilitation in Major Abdominal Surgery—Rationale, Modalities, Results and Limitations

**DOI:** 10.3390/medicina61050908

**Published:** 2025-05-17

**Authors:** George Andrei Popescu, Dana Galieta Minca, Nader Mugurel Jafal, Cristian Valentin Toma, Sorin Tiberiu Alexandrescu, Radu Virgil Costea, Catalin Vasilescu

**Affiliations:** 1 Faculty of Medicine, Carol Davila University of Medicine and Pharmacy, Bulevardul Eroii Sanitari 8, Sector 5, 050474 Bucharest, Romania; george-andrei.popescu@drd.umfcd.ro (G.A.P.); dana.minca@umfcd.ro (D.G.M.); mugurel.jafal@drd.umfcd.ro (N.M.J.); cristian.toma@umfcd.ro (C.V.T.); catalin.vasilescu@umfcd.ro (C.V.); 2 Department of Hepato-Bilio-Pancreatic Surgery, Emergency University Hospital Bucharest, Splaiul Independentei 169, Sector 5, 050098 Bucharest, Romania; 3 Department of Public Health and Management, Dr. Leonte Anastasievici Street 1-3, Sector 5, 050463 Bucharest, Romania; 4 Department of Anaesthesiology and Intensive Care, Emergency University Hospital Bucharest, Splaiul Independentei 169, Sector 5, 050098 Bucharest, Romania; 5 Department of Urology, “Prof. Dr. Theodor Burghele” Clinical Hospital, Soseaua Panduri 20, Sector 5, 050659 Bucharest, Romania; 6 2nd Department of Surgery, Emergency University Hospital Bucharest, Splaiul Independentei 169, Sector 5, 050098 Bucharest, Romania; 7 Department of Surgery, Fundeni Clinical Institute, Soseaua Fundeni 258, Sector 2, 022328 Bucharest, Romania

**Keywords:** prehabilitation, abdominal surgery, enhanced recovery after surgery (ERAS), surgical stress, physiological reserve, nutritional support, exercise intervention

## Abstract

Recent evidence revealed that an adequate preoperative physiological reserve is crucial to overcome surgical stress response. Consequently, a new concept, called prehabilitation, emerged, aiming to improve the preoperative functional reserve of patients who will undergo major abdominal surgery. During the interval between diagnosis and surgery, a multimodal approach consisting of physical exercise and nutritional and psychological support could be employed to enhance physiologic reserve. Physical activity interventions aim to improve aerobic capacity, muscle strength and endurance. Nutritional support addressing malnutrition and sarcopenia also contributes to the achievement of the above-mentioned goals, particularly in patients undergoing cancer-related procedures. Psychological interventions targeting anxiety, depression and self-efficacy, as well as risk behavior modification (e.g., smoking cessation) seem to enhance recovery. However, there is a lack of standardization regarding these interventions, and the evidence about the impact of this multidisciplinary approach on the postoperative outcomes is still contradictory. This narrative review focuses on the physiological basis of surgical stress response and on the efficacy of prehabilitation, reflected mainly in the length of hospitalization and rates of postoperative complications. Multidisciplinary collaboration between surgeons, nutritionists, psychologists and physiotherapists was identified as the key to the success of prehabilitation programs. Synergizing prehabilitation and ERAS protocols significantly improves short-term surgical outcomes. Recent well-designed, randomized clinical trials revealed that this approach not only enhanced functional reserve, but also decreased the rates of postoperative complications and enhanced patient’s overall quality of life, emphasizing the importance of its implementation in routine, elective, surgical care.

## 1. Introduction

Major surgical procedures are frequently associated with prolonged postoperative pain, cardiopulmonary complications and surgical site infections, leading to extended recovery periods, primarily as a result of an exacerbated body’s stress response. The physiological response to surgical stress is considered to be an innate survival mechanism designed to restore the body’s homeostasis as quickly as possible. This response, induced by trauma-related immunologic and neuro-hormonal changes, activates multiple biological pathways that impact recovery, having a major impact on metabolism. These pathways induce the catabolism of glycogen, fat and proteins, releasing glucose, free fatty acids and amino acids, which are substrates for the production of inflammatory phase proteins that contribute to healing and immune response [1,2]. Nevertheless, an inappropriate, exaggerated or prolonged stress response can lead to negative outcomes, such as increased protein catabolism [3,4]. This exacerbated catabolism of proteins leads to loss of muscle tissue, which is a short- and long-term burden for functional recovery [5]. To enhance muscle mass before surgery and/or restore it postoperatively, adequate nutrition and physical exercise are essential. It was hypothesized that the optimization of these factors may contribute to enhanced postoperative recovery. Thus, over the past several decades, various management strategies and protocolized care pathways have been developed to decrease the rates of postoperative complications and shorten the recovery time following surgery. While no single intervention can completely fulfill these goals, adopting multimodal approaches (such as exercise, opioid-sparing pain management plans, epidural analgesia, minimally invasive approaches, tailored fluid management, ileus-prevention interventions, anti-emetic prophylaxis and nutritional and psychological optimization) has been shown to enhance physiological function and resilience, alleviate adverse outcomes, accelerate recovery and reduce healthcare costs, providing a comprehensive solution to minimize the rates of postoperative complications [6,7]. The current medical approach is to intervene pre-, peri-, and postoperatively to modulate surgical stress response in order to minimize its negative effects, while maintaining the natural goal of achieving the homeostasis of the body. In this narrative review, we present the physiologic pathways of surgical stress response, as well as the therapeutic approaches that can interfere with these pathways in order to avoid the development of an inappropriate surgical stress response, which can lead to postoperative complications and longer recovery periods. The mechanisms of action, the components of each multimodal intervention, as well as their synergistical effects are analyzed to assess which patients can derive the greatest benefits from their implementation. Furthermore, the results of the most relevant clinical trials are summarized and discussed to better understand the impact of prehabilitation modalities on measurable outcomes, as well as to explain the inconclusive results of many small-sample-size studies, which used different types of physical activities. The limitations of this study, as well as of the current trials, are also pointed out. Thus, this review offers a framework of the current interventions used in the multimodal prehabilitation addressed mainly to the high-risk patients who underwent major gastrointestinal operations for malignancies (especially colorectal cancer) and make recommendations regarding the subgroups of patients who might achieve the greatest benefits from personalized preoperative interventions (physical, nutritional and psychological).

## 2. Mechanism of Surgical Stress

**Figure 1:**
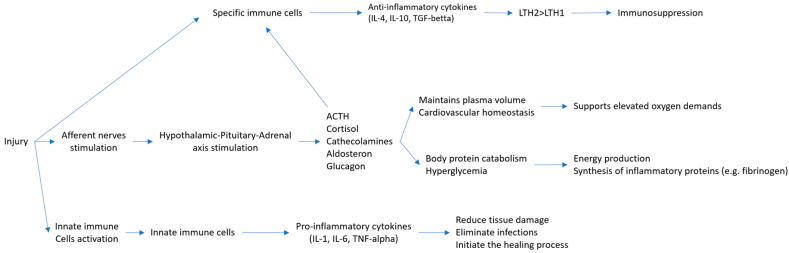
Neuro-endocrine and immunologic mechanisms involved in surgical stress response.

Surgery activates the body’s normal physiologic response known as the surgical stress response. Surgical injury activates the hypothalamic–pituitary–adrenal axis and the sympathetic nervous system by stimulating afferent nerve pathways and cytokines [8,9]. This generates integrated endocrine, hemodynamic and immune responses to restore the body’s homeostasis (state of dynamic equilibrium) (Figure 1).

The endocrine response alters intermediate metabolism by catabolizing proteins contributing to energy production and initiates the synthesis of inflammatory proteins (e.g., fibrinogen), which promote wound healing. Clinically, the endocrine response leads to hyperglycemia, increased blood levels of urea and creatinine and even sarcopenia [1].

The hemodynamic response maintains cardiovascular and intravascular fluid homeostasis providing adequate oxygenation of the tissues, as there is an increased oxygen demand due to surgical stress. Clinically, the hemodynamic response can induce hypertension, tachycardia, decreased urinary output and even oedema [2].

The immune response maintains an adequate balance between pro- and anti-inflammatory cytokines, which act both locally and systemically to initiate the healing process and, at the same time, to reduce tissue damage, eliminate pathogenic microorganisms and avoid infection [9,10].

Following surgery, the body responds physiologically by catabolizing energy sources (glycogen, reserve lipids and proteins) that aid energy production and facilitate the precursors of the processes responsible for postoperative recovery. Although glycogen and body fat are energy reserves, when they are depleted, the body resorts to protein catabolism in an attempt to restore homeostasis. This leads to a vicious circle because the use of protein as an energy source implies a decrease in the functions of the viscera of which they are part [11,12,13,14]. Therefore, a patient with suboptimal energy reserves preoperatively (e.g., malnutrition) does not have the resources to sustain the healing process. In light of this evidence, some traditional practices with regard to preoperative preparation have been shown to include unnecessary elements that only accentuate the surgical stress response [15,16]. Thus, fasting the night before surgery has been proven as a stressor that increases the body’s efforts to return to homeostasis and thus amplifies the postprocedural catabolic response. Many institutions continue to enforce these practices regarding fasting, although new anesthesia and intensive care guidelines recommend the consumption of food up to 6 h and clear liquids up to 2 h before surgery [17,18,19].

## 3. Functional Capacity and Physiological Reserve

Functional capacity is the body’s ability to use the pulmonary, locomotor and cardiovascular systems in order to approach and fulfil the activities of daily life. To perform daily tasks and exercise, a minimum (basal) threshold of physiological capacity is necessary. Under the influence of certain stressors (e.g., trauma, surgery, etc.), the physiological demands to achieve the homeostasis of the body are higher. Thus, the organism should have a physiological capacity higher than the basal threshold in order to cope with the stressor factor. This potential capacity of a cell, tissue or organ system to function beyond its basal level in response to alterations in physiologic demands is called physiological reserve [20]. For a surgical patient, oxygen requirements can increase by up to 50% in the postoperative period, mainly to maintain the adequate function of the liver and muscles [21,22,23]. Many studies demonstrated that the poor capacity of the cardiopulmonary system to deliver oxygen under stressful conditions leads to an impairment in achieving homeostasis following surgery [21,23,24,25]. Thus, the functional independence of patients with limited physiological reserves can be jeopardized after an operation, increasing the risks of postoperative complications and prolonged recovery period. Therefore, an adequate threshold of physiological capacity is required to be intact to safely overcome the postoperative period [26,27,28,29]. The achievement of post-surgical homeostasis depends on the balance between the patient’s physiological reserve and physiological capacity on one hand, and the extent of the surgical injury on the other hand.

The physiological reserve is influenced by the interdependent relationship between the physical, nutritional and psychological reserves.

From a cellular point of view, the optimal physiological reserve can be understood as a surplus of metabolic capacity over the basal level, which can quickly sustain an additional metabolic requirement. Disease and aging (frailty) reduce or eliminate this surplus and thus, with a diminished physiological capacity, the individual ends up having an unsatisfactory response to surgical stress [30,31,32].

However, the optimal modality to assess the functional capacity of a patient is not universally accepted. It can be assessed either subjectively (grading the functional capacity in poor, moderate or good) or objectively (e.g., cardiopulmonary exercise testing—CPET, scores on the Duke activity status index—DASI questionnaire, or serum levels of N-terminal pro-B-type natriuretic peptide—NT pro-BNP) [25]. In a recent multicenter, international, prospective cohort study, Wijeysundera et al. [25] compared the ability of these methods to predict morbidity and mortality in 1401 patients who underwent major abdominal, pelvic or orthopedic surgery. They found that lower DASI scores can accurately predict 30-day death or myocardial infarction/injury, increased levels of NT pro-BNP predict 30-day death or myocardial injury, as well as 1-year death, while lower peak oxygen consumption predicts postoperative complications. However, the subjective assessment was not able to predict any outcomes. These results support the need for an objective evaluation of functional capacity before surgery, as the subjective evaluation failed to reliably predict postoperative outcomes.

Low energy reserves imply a low physiological reserve, which leads to an impaired physiological response following surgery, which is observed in the frailty phenomenon. Thus, in these patients, the physiological response to surgical stress negatively affects the convalescence process. By comparison, patients with an optimal physiological reserve have resources to reach homeostasis within a shorter period of time [33]. Preoperative status with an optimal protein reserve allows the postoperative maintenance of the inflammatory response to surgical stress without diminishing basic functions. Thus, Malietzis et al. [34] evaluated the impact of body composition profile on the short-term and long-term outcomes of 805 patients operated on for colorectal cancer. After a median follow-up period of 47 [24.9–65.6] months, they found that myopenia was an independent predictor of both disease-free survival (DFS) (HR—1.53, 95% CI: 1.06–2.39; *p* = 0.041) and overall survival (OS) (HR—1.70, 95% CI: 1.25–2.31; *p* < 0.001). In addition, myosteatosis was associated with longer primary hospital stays (*p* = 0.034), while myopenic obesity was associated with increased morbidity (*p* = 0.019) and mortality (*p* < 0.001) rates at 30 days. Lawrence et al. found that patients with preoperative sarcopenia, who underwent major abdominal surgery, felt the surgical stress more acutely, because the last energy reserve of the body was reduced. Thus, a hypoproteinemic patient experiences a decrease in skeletal and intestinal muscle function, which is clinically translated postoperatively as a longer period of immobilization, impaired breathing and intestinal tract disorders. All this can lead to complications that increase the period of hospitalization and decrease the quality of life, despite well-performed surgery [35,36].

In malnourished patients, it has been observed that if the inflammatory and immune responses generated after surgical stress are not potent enough to restore homeostasis, the risk of postoperative complications increases, leading to prolonged hospitalization, more frequent readmissions, increased mortality and hospitalization costs [37,38,39]. To analyze the risk of postoperative mortality, Vaid et al. [39] evaluated the preoperative data of 202,741 (68%) patients operated on in the USA in 2005–2008, and 94,060 (32%) patients operated on 2009 for pancreatic pathology (by open surgery), colorectal pathology (by open and laparoscopic approaches), parietal defects (such as ventral, umbilical and inguinal hernias) and gallbladder pathology. They found that impaired functional status and weight loss were independent factors associated with postoperative mortality and incorporated these parameters in a preoperative mortality predictor score (PMP score), which had 98.6% accuracy (*p* < 0.05) in predicting postoperative mortality.

The first interventions that aimed to interfere with the pathophysiologic mechanisms involved in the development of an inappropriate surgical stress response were implemented in the perioperative period and led to the development of the enhanced recovery after surgery (ERAS) concept.

## 4. Enhanced Recovery After Surgery

The concept of “enhanced recovery” (ER), initially introduced by Kehlet, is founded on the idea that traditional perioperative practices, such as preoperative fasting, liberal fluid administration during surgery and the use of nasogastric tubes along with opioid-centered pain management post-surgery, should be re-evaluated and adjusted according to the latest evidence-based medicine [7,40].

Over time, the concept evolved into “enhanced recovery after surgery” (ERAS), a multimodal, perioperative care pathway aimed at reducing stress response throughout a patient’s surgical experience [15]. The objective of this approach is to maintain optimal bodily composition and organ function perioperatively, thereby promoting a quicker recovery. Several factors contribute to prolonged hospital stays following otherwise uncomplicated major abdominal surgery, including the requirement for parenteral analgesia, continued intravenous fluid administration due to persistent gut dysfunction and extended periods of bed rest caused by limited mobility. All these factors significantly delay recovery, underscoring the importance of preoperative interventions that enhance functional capacity and reduce surgical stress [41].

The implementation of ERAS protocols has had a significant impact on the prognosis and evolution of patients undergoing abdominal surgery. By optimizing perioperative care with a multidisciplinary approach, ERAS has been shown to reduce complications, shorten hospital stays and improve recovery times [42]. In particular, the use of these protocols has led to a decrease in postoperative morbidity and mortality, as well as reduced healthcare costs [42,43]. Single-center studies have demonstrated that ERAS protocols, compared to conventional care, result in fewer complications, such as pulmonary, infections and cardiovascular events, as well as lower readmission rates [44]. Furthermore, there are numerous multicenter, international cohort studies, as well as meta-analyses attesting to the effectiveness of ERAS protocols in major abdominal surgery [45,46,47,48]. Efficiency means the decrease in the length of hospital stay, a reduction in complication rates and a reduction in costs of hospitalization [44,49].

Thus, Noba et al. [45] conducted a systematic literature review and meta-analysis of studies published between 2008 and 2019, comparing the effect of ERAS protocols versus standard care on liver surgery patients. Several outcomes were assessed, such as cost of hospitalization, duration of hospitalization, complications, readmission and mortality rates. The cohort included 3739 patients (1777 in ERAS and 1962 in the standard care group). The results showed a lower hospital cost in the ERAS group compared to the standard care group (SMD = −0.98; CI, −1.37 to −0.58; *p* < 0.0001). Length of hospitalization was 2.22 days shorter in the ERAS group (MD = −2.22; CI, −2.77 to −1.68; *p* < 0.00001) compared to the standard care group. Fewer patients in the ERAS group had complications (RR, 0.71; 95% CI, 0.65 to 0.77; *p* < 0.00001). However, there was no significant difference in readmission rates between ERAS and standard care groups (RR, 0.94; 95% CI, 0.70 to 1.26; *p* = 0.68) and also no significant difference in mortality rates (RR, 0.67; 95% CI, 0.30 to 1.49; *p* = 0.33).

Greco et al. [46] conducted a meta-analysis on a total of 2376 patients undergoing elective colorectal surgery, seeking differences between the group that benefited from the application of ERAS protocols and the one that did not. They observed a significant reduction in overall morbidity (RR = 0.60, 95% CI 0.46–0.76), particularly concerning non-surgical complications (RR = 0.40, 95% CI 0.27–0.61), but the reduction in surgical complications was not significant (RR = 0.76, 95% CI 0.54–1.08). Hence, the ERAS group had a shorter hospital stay without increasing the readmission rate (WMD = −2.28 days, 95% CI −3.09 to −1.47).

Pecorelli et al. [47] conducted (between 2012 and 2014) a prospective study on 347 patients undergoing elective bowel resection and an ERAS protocol. The ERAS protocol included 23 care elements. Patients were compliant with a median of 18 items (IQR 16–20). A significant association was demonstrated between adherence to the ERAS protocol and length of hospitalization (11% reduction for each additional element, *p* < 0. 001), as well as a 30-days morbidity rate (OR 0.78 for each additional element, *p* = 0.001). Minimally invasive surgery (OR 4.32, *p* = 0.001), early mobilization from bed (OR 2.25, *p* = 0.021) and early cessation of i.v. fluid infusion (OR 2.00, *p* = 0.013) were associated with reduced morbidity and complication severity.

The ERAS International Study Group [48] conducted an international multicenter study between 2008 and 2013, aiming to analyze the postoperative effects of ERAS protocols on patients with elective colorectal cancer resections. The study enrolled 2352 patients from 13 centers in 6 countries (1509 with colonic resections and 843 with rectal resections). The mean length of hospitalization was 6 days, and its shortening was positively influenced by the minimally invasive approach (OR = 0.83, *p* < 0.001), compliance to protocol (OR = 0.88, *p* < 0.001), preoperative carbohydrate and fluid loading (OR = 0.89, *p* = 0.001) and total intravenous anesthesia (OR = 0.86, *p* < 0.001).

However, most ERAS guidelines in abdominal surgery include elements of anesthesia management, surgical approach, postoperative care and discharge plans, but more than half of ERAS protocols lack recommendations regarding the preoperative optimization of physiologic capacity and reserve [50].

**Figure 2:**
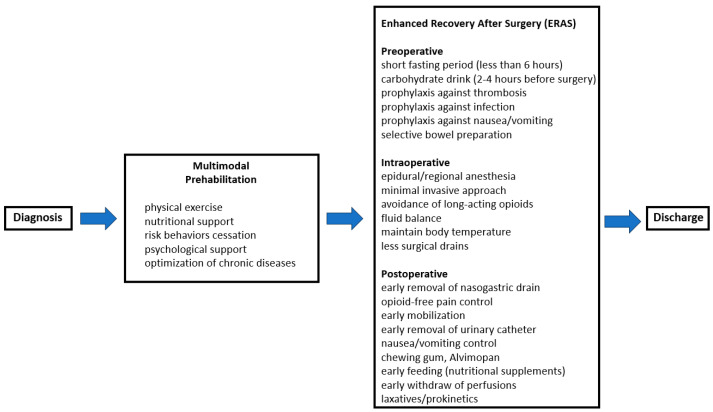
Pathway of the surgical patient to enhance recovery.

For this reason, multidisciplinary management aiming for the optimization of the preoperative physiological reserve is a distinct approach from the ERAS protocols called prehabilitation, and its implementation was associated with encouraging results for patients scheduled for major abdominal surgery, as has already been mentioned [2] (Figure 2). Thus, prehabilitation programs (defined as the enhancement of the patient’s preoperative conditions) encompass pre-admission information; education and counseling for the patient; cessation of harmful behaviors, such as smoking and alcohol consumption; physical exercise; adequate preoperative nutrition and anemia management when applicable as well as psychological support [51,52]. By addressing the functional capacity preoperatively, the postoperative risk is attenuated [53].

## 5. Principles of Prehabilitation

### 5.1. The Concept of Prehabilitation

Our society has become increasingly sedentary, with many opting for cars instead of walking or cycling, spending long hours in front of screens and creating environments that discourage physical activity. Despite this, a significant body of evidence highlights the numerous health benefits of physical fitness, suggesting that those who are fitter tend to experience better health outcomes across various settings [54]. This growing body of evidence underscores the importance of maintaining physical fitness for improved quality of life and disease prevention [55,56].

Furthermore, poor preoperative physical condition has been identified as a significant risk factor for serious postoperative complications and extended disability after major abdominal surgical procedures [57]. For this reason, it was hypothesized that by optimizing the patient’s physical condition through interventions such as exercise and nutrition, it is possible to reduce the risk of complications and speed up the recovery process after surgery [58]. Thus, the period between diagnosis and operation offers a valuable opportunity to enhance the body’s physiological reserve in preparation for surgery, with the goal of improving surgical outcomes and facilitating faster recovery. This multidisciplinary approach aiming to improve preoperatively the physiological reserve is known by various names, the most commonly recognized being prehabilitation. However, in the literature, it can also be referred to as “Fit 4 Surgery”, “Fit 2 Fight” or “Better in—Better out”. All these terms refer to the same revolutionary concept aimed at optimizing a patient’s condition prior to surgery to improve surgical outcomes and accelerate recovery [53].

Although no clear and concise definition exists, prehabilitation is generally described as a process within the continuum of care that occurs between the time of diagnosis and the moment of surgery (Figure 2). It encompasses physical, nutritional and psychological assessments aimed at establishing the baseline functional level, identifying potential impairments and implementing interventions that allow the increase in physiological reserve [59]. Thus, the ultimate goal of prehabilitation is to reduce the incidence and/or severity of future impairments, thereby improving patient outcomes and enhancing recovery [60]. Specifically, prehabilitation targets modifiable risk factors to improve the perioperative functional capacity, especially in elderly, frail patients undergoing elective surgery for malignancies [61,62]. Because prehabilitation interventions are recommended mainly to elderly patients with different physiological impairments, the prehabilitation protocol should be tailored according to the physical, nutritional and psychological conditions of the patient, taking also into account the comorbid conditions of the patient.

**Figure 3:**
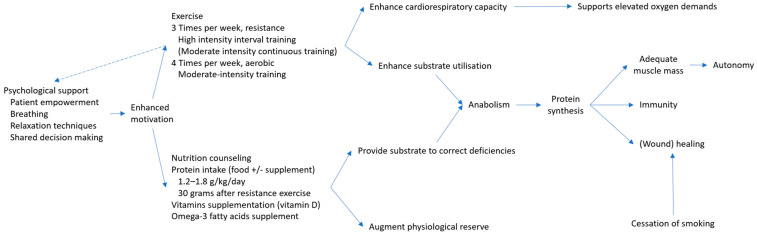
Synergistical effects of multimodal prehabilitation methods.

Thus, to date, prehabilitation is a multimodal intervention based on four pillars: physical activity, nutritional support, correction of harmful behaviors and comorbidities and psychological support [63]. These interventions act synergistically, contributing to a better physiological reserve (Figure 3). As such, psychological support aiming to provide patient empowerment and decrease anxiety or/and depression will enforce the motivation for training and eating [53]. Exercise and dietary protein intake produce independent and additional effects on anabolism and muscle protein synthesis [64]. Furthermore, smoking cessation 4–8 weeks before surgery led to a decrease in pulmonary complications at 25–50% compared to current smokers and significantly improved wound healing [65].

### 5.2. Physical Activity

Prehabilitation programs aim to enhance aerobic fitness and reduce postoperative complications by focusing on key exercise parameters. These parameters include the mode, intensity, frequency, duration and volume of exercise [66]. The identification of the activity type in which the patient participates can offer the therapist valuable insights into the patient’s preferences for exercise modalities within both clinical and community contexts [67].

Prehabilitation programs often integrate a range of exercises, focusing on both aerobic and resistance (strength) training to enhance physical fitness and lower postoperative risks.

Aerobic exercises are recommended to enhance cardiovascular capacity and endurance. The goal of these exercises is to improve blood circulation, lung function and tissue oxygenation, which benefits post-surgical recovery. Examples of recommended activities include: brisk walking or light jogging—which helps increase cardiovascular endurance without putting excessive pressure on joints; cycling (whether on a stationary or regular bike)—which is excellent for strengthening leg muscles and improving circulation; and swimming—which is a low-impact exercise, but suitable for those with joint or muscle issues. The duration and intensity of aerobic exercises should be personalized. However, a common goal is to achieve approximately 150 min of moderate aerobic activity per week, spread over 3–5 sessions of 30–50 min each [68].

The guidelines also encourage resistance exercises to build muscle strength and prevent muscle mass loss. This is particularly important, as patients who lose muscle mass before surgery may face a more challenging recovery. Examples of resistance exercises include: weightlifting light-to-moderate weights, which are recommended for muscle toning, though training should be adapted to the patient’s tolerance and fitness level, and elastic-band exercises, which allow variable resistance, making them suitable for patients of all ages and fitness levels. Furthermore, bodyweight exercises (e.g., squats, push-ups and lunges) are highly effective for strengthening muscles. These exercises are versatile and can be modified to avoid overloading the joints. Two to three resistance training sessions per week are recommended, with a gradual increase in weights or resistance to ensure progressive muscle development [68].

Flexibility and mobility exercises are essential for preventing stiffness and maintaining a normal range of motion, which can reduce postoperative muscle discomfort and improve mobility. Exercises include: static stretching—stretching major muscle groups (quadriceps, hamstrings, back muscles and arm muscles) helps maintain flexibility; dynamic stretching—controlled, gradual movements are recommended to increase joint mobility; and yoga, which is helpful for muscle strengthening and relaxation, as well as for improving balance and coordination. Additionally, yoga helps reduce stress, which can be beneficial in the preoperative period [69].

The addition of cross-training and inspiratory muscle training can further boost endurance and respiratory function, which is particularly beneficial for cancer patients preparing for surgery [70]. Incentive spirometry is also an important and useful tool to optimize preoperatively the strength of respiratory muscles.

Exercise intensity plays a critical role in enhancing physical fitness. Prehabilitation programs often categorize exercise intensity into three levels: low, moderate and high. Each intensity level is designed to meet different patient needs and physical conditions. Cancer prehabilitation studies primarily used moderate-to-vigorous exercise intensities, as they have been shown to produce significantly greater gains in aerobic fitness than low-intensity activities. A prehabilitation program consisting of aerobic exercises at a moderate intensity along with periodic high-intensity intervals significantly improved cardiovascular function [58].

Exercise volume, measured by the combination of intensity, duration and frequency, can be achieved through either short, high-intensity sessions or longer, moderate-intensity sessions, offering flexibility in structuring effective workouts. As suggested by Weggemans et al., the higher exercise volumes, the better health benefits [71]. Exercise volume is initially increased by extending the frequency and duration, while later stages prioritize intensity adjustments to sustain or enhance the workout’s overall impact [71]. A study conducted in 2022 revealed that in patients undergoing major abdominal surgery, preoperative, high-intensity interval training (HIIT) significantly enhances aerobic capacity, even with a brief training duration (14 sessions over 4 weeks) [72]. One of the most comprehensive and well-documented prehabilitation programs was proposed by van Rooijen et al. in 2019, who initiated the first international, multicenter, randomized controlled trial to evaluate the impact of a 4-week multimodal prehabilitation program on functional capacity and postoperative complications in patients operated on for colorectal cancer [73]. According to the study protocol, the exercise program is decided by a kinesiologist and sport physician, who initially evaluate the patient’s mobility and capacity to exercise using a cardiopulmonary exercise test (CPET). With the purpose of standardization, the authors employed an exercise program consisting of an interval training duration in the range of 28–32 min, which included 4 min of warm-up at a moderate intensity, 4 intervals of HIIT (2–3 min) alternated with 4 intervals of moderate-intensity exercise (4 min). The workload is set at 90% of the peak wattage attained by the CPET for high-intensity exercise and at 30% for moderate-intensity exercise. If the patient is unable to complete the 4 intervals of HIIT for at least 2 min, the intensity is reduced in steps of 10% until the patient can complete the 4 bouts of minimum 2 min [73]. Furthermore, strength training consisted of 2 series of 10 repetitions of 6 exercises targeting all major muscle groups. The exercise dosing is adjusted according to the attainability of the last bout with 10 repetitions [73]. Patients should receive guidance about how to conduct aerobic exercises at home, mainly by walking and/or cycling at least 30 min a day.

An alternative to HIIT as part of a 4-week multimodal prehabilitation program is moderate-intensity continuous training (MICT). RCT revealed that both HIIT and MICT enhanced preoperative functional capacity, with no difference between groups (*p* = 0.753) [74].

Preoperative oncologic therapies lead to a significant decline in physical fitness, increasing the risk of poor postoperative outcomes. In such patients, prehabilitation interventions seem to be crucial to improve postoperative recovery. The impact of a structured, six-week exercise prehabilitation program was evaluated by West et al. in patients with rectal cancer who received neoadjuvant chemoradiotherapy (NACRT) [75]. The prehabilitation group experienced a significant improvement in peak VO2 consumption (+2.12 mL/kg/min, 95% CI 1.34–2.90; *p* < 0.0001) while the control group did not (−0.7 mL/kg/min, 95% CI −1.66–0.37; *p* = 0.204). This improvement in physical fitness suggests that prehabilitation before surgery may mitigate the decline in fitness due to NACRT, which can be critical for reducing postoperative complications.

Regarding the molecular mechanisms involved in reducing surgical stress response due to physical exercise, recent observations revealed that exercise training modulates inflammatory markers toward an anti-inflammatory state and reduced pro-inflammatory markers [76,77]. A more recent study evaluated the impact of physical exercise on molecular changes in patients with gastrointestinal cancers [78]. Transcriptomic analyses revealed a shift toward an anti-inflammatory and anti-tumorigenic phenotype in the serum of patients who underwent exercise prehabilitation, compared to the control group. Furthermore, following exercise, the authors found significant alterations in pathways such as steroid hormone biosynthesis, arachidonic acid metabolism and starch/sucrose metabolism, all these pathways being involved in tumor progression [78]. These findings may explain not only the improvement in the short-term outcomes of prehabilitated patients, but also the potential favorable impact of exercise training on the long-term outcomes of patients operated on for malignancies.

Although exercise can increase muscle mass as a result of enhanced substrate utilization, this result cannot be attained unless adequate nutritional support is offered.

### 5.3. Nutritional Support and Correction of Comorbid Conditions

The success of surgery is not solely reliant on the technical proficiency of the surgical team, but is also influenced by how patients respond to the physiological stressors imposed by the procedure. As such, providing nutritional support preoperatively, rather than reactively during the perioperative period, is critical. Thus, the assessment of nutritional status plays a critical role during the prehabilitation phase. It is recommended to evaluate all patients for the risk of malnutrition, obesity or other nutritional deficiencies. Morphofunctional assessment is widely used at present to evaluate body composition and muscle strength in patients who will undergo multimodal prehabilitation interventions. Beyond the nutritional screening tools used for the assessment of nutritional status (such as weight loss in 3–6 months, serum albumin level, BMI—body mass index, NRS—nutritional risk screening, SGA—subjective global assessment, Duke University Pre-Operative Nutrition Score—PONS, and MUST—malnutrition universal screening tool [79]), nowadays, for the evaluation of body composition, methods such as bioelectrical impedance analysis (BIA) and dual-energy X-ray absorptiometry (DEXA) are widely used. Another simple and reliable method to evaluate muscle strength and sarcopenia is handgrip strength (HGS). These methods may better classify patients at baseline. According to the results of the preoperative assessment, adjustments should be tailored to each patient’s individual needs, based on their initial nutritional status. The prehabilitation phase focuses on improving the patient’s metabolic reserve, muscle mass and overall nutritional state. This includes addressing nutritional deficiencies, optimizing protein intake and managing metabolic alterations to reduce catabolism and facilitate recovery. Although in adult, healthy patients the daily requirement of proteins is about 0.8 g/kg/day, during the perioperative period, the protein requirement is at least 1.2 g/kg/day [80]. In malnourished patients, a balanced diet containing 1.5–1.8 g/kg/day of proteins is recommended in the preoperative period. It is preferable to divide this amount of protein into three equal portions. Furthermore, in patients performing resistance training, to achieve enhanced muscle mass, a supplement of 30 g of protein is recommended within one hour of training to make use of the “anabolic window” (the moment at which muscle protein synthesis is the highest) [81]. Malnutrition, whether resulting from inadequate intake nutrition or metabolic disturbances, can significantly impair healing, increase the likelihood of postoperative complications and extend hospitalization [82]. Thus, Simonsen et al. [83] conducted a meta-analysis of 29 studies enrolling 7176 patients undergoing surgery for gastrointestinal cancers between 2004 and 2017, to investigate the relationship between sarcopenia and postoperative complications. The results revealed that sarcopenia is linked to a significantly higher risk of both major (RR 1.40; 95% CI 1.20–1.64; *p* < 0.001) and total (RR 1.35; 95% CI 1.12–1.61; *p* = 0.001) postoperative complications. Thus, the guidelines emphasize the importance of early nutritional screening, individualized dietary support and the use of oral nutritional supplements or enteral nutrition when appropriate [5]. According to the ESPEN guideline, oral nutritional supplements are recommended to all patients who do not meet their energy needs from normal food, irrespective of their nutritional status. Furthermore, patients with severe nutritional risk should receive nutritional therapy prior to major surgery, for a period of 7–14 days, even if operations (including those for cancer) have to be delayed [84]. Furthermore, oral/enteral nutrition is preferred, parenteral nutrition being reserved only for patients whose energy requirements cannot be adequately met by enteral nutrition [84]. Preoperative nutritional optimization is essential, as it has been shown to minimize complications such as infections, delayed wound healing and muscle wasting, especially in frail patients operated on for digestive malignancies [85].

Nutritional prehabilitation aims to enhance nutrient stores and metabolic capacity before surgery, providing a buffer against the catabolic effects of surgery or critical illness. Unlike acute nutritional supplementation, which primarily addresses immediate deficiencies, nutritional prehabilitation is a proactive approach that begins at the time of surgery planning, engaging the patient early and extending throughout the perioperative and postoperative phases to ensure sustained benefits.

The risk of malnutrition is particularly pronounced in elderly patients with gastrointestinal cancers, more than 50% of them having baseline malnutrition [86]. Surgery and critical illness lead to significant protein catabolism, which should be fully understood and addressed. Ensuring adequate protein intake, regardless of overall caloric intake, is essential for preserving lean muscle mass, muscle function and mitigating the risk of frailty, particularly in elderly patients [87,88]. Thus, according to the ESPEN guidelines on clinical nutrition in surgery, oral nutritional supplements should be administered preoperatively to all malnourished and elderly people with sarcopenia undergoing major abdominal surgery for cancer [84]. Oral nutritional supplements should comprise a standard, fully balanced, non-disease-specific formula, which may be used as a sole source of nutrition. Immune-modulating, oral, nutritional supplements, including arginine, omega-3 fatty acids and nucleotides, may be preferred, although there is no clear evidence about their enhanced benefits over standard, oral, nutritional supplements [84]. However, a secondary analysis of the PREHAB RCT concluded that achieving optimal protein intake was challenging, being significantly more often accomplished in the prehabilitation group. Both dietary counseling and supplements are recommended to improve protein intake [89].

However, it is not only malnutrition that poses a challenge to the postoperative outcomes of surgical patients. Obesity, a truly current global issue, significantly affects the success of surgery. Obesity is associated with a range of complications, including delayed recovery, increased risk of postoperative infections, impaired wound healing and heightened metabolic stress [90]. The prehabilitation nutritional recommendations highlight the importance of managing obesity and improving nutritional status before surgery to reduce these risks and enhance recovery outcomes. The primary concern with obesity is its impact on muscle mass and metabolic function. Obesity can lead to sarcopenic obesity, where individuals have excess fat but low muscle mass, further compromising postoperative recovery and functional outcomes [91,92]. The current evidence suggests that preoperative interventions should focus on improving body composition, particularly preserving or increasing muscle mass through nutritional optimization and exercise. A trimodal preoperative intervention statistically increased fat free mass (0.9 kg, *p* = 0.017) and appendicular skeletal muscle mass (0.5 kg, *p* = 0.007) compared to the standard of care, being also associated with a significant increase (27%) in muscle strength (*p* =< 0.001) in patients operated on for colorectal cancer [89].

In prehabilitation programs for obese patients, the goal is not only to manage caloric intake, but also to ensure adequate protein intake to support muscle mass and prevent catabolic muscle loss. This may involve dietary adjustments, weight management strategies and physical activity interventions tailored to the individual’s capabilities and limitations. Beyond weight loss, the prehabilitation phase seeks to optimize overall physical fitness, reducing surgical risks and improving postoperative outcomes [93,94]. Studies have shown that preoperative weight management can improve surgical outcomes, particularly by reducing postoperative complications and enhancing functional recovery. Furthermore, a randomized, controlled feasibility trial for perioperative nutrition among patients undergoing major abdominal surgery for malignancies revealed that perioperative nutrition was associated with the preservation of quality of life (QoL) in the postoperative period, compared to the placebo [95].

These benefits of nutritional interventions are even more pronounced when combined with structured exercise programs (mainly based on HIIT and strength training) that increase cardiovascular and muscular endurance [96]. These interventions act synergistically, since nutrition supplements provide substrate to correct nutritional deficiencies and resistance exercises enhance substrate utilization, leading to anabolism and protein synthesis.

A patient who is to undergo surgery but suffers from various pathologies may also present micronutrient deficiencies, such as iron, zinc, magnesium, potassium and sodium. These deficiencies can impair immune function, hinder the ability to recover and affect tissue healing. In patients with gastrointestinal diseases, deficiencies in fat-soluble vitamins (A, D, E, K) may lead to issues such as vision problems, bone weakness, neurological dysfunction and coagulation issues. Inadequate hydration can complicate the management of intraoperative fluids [97]. Interventions to correct these deficiencies may include oral supplementation, and in more severe cases, enteral or parenteral nutrition tailored to the patient’s needs.

Moreover, the management of preoperative anemia is recommended, as it is associated not only with detrimental short-term outcomes, such as higher rates of postoperative complications, but also with reduced overall and disease-free survival rates in patients with rectal cancer [98,99]. Of course, the preoperative optimization of comorbid conditions of the patients is tremendously important and should be integrated in the continuum of care.

### 5.4. Harmful Behaviors Management

To achieve optimal health in preparation for surgery, it is essential not only to engage in regular physical activity and improve nutritional status, but also to eliminate harmful behaviors that can further compromise the patient’s outcomes. The most common behaviors include smoking (and more recently vaping, but less studies have been published about vaping), alcohol consumption and drug addiction.

Smoking poses a significant perioperative risk by compromising pulmonary, cardiac and immune functions, which affects up to 25% of surgical patients and leads to an increased likelihood of complications, such as pneumonia, cardiac events and poor wound healing. Schmid et al. [100] conducted a study to assess the influence of perioperative smoking on morbidity and mortality. Their results showed that current and former smokers had more complications compared with nonsmokers (14.9% and 14.6% vs. 12.5%, *p* < 0.001). Also, pulmonary, neurologic and wound complications were most common in current smokers, whereas thromboembolic phenomena, sepsis/shock and renal failure were seen most frequently among former smokers (all *p* < 0.001). Furthermore, smokers had higher rates of reintervention (7.5%) and readmission (11.1%).

The general recommendation is to stop smoking 4–8 weeks prior to surgery. All smokers should receive preoperative support to quit, as measurable physiological benefits begin within minutes of the last cigarette. This support should ideally combine counseling and tailored nicotine replacement therapy, facilitated through access to a cessation service [101].

Chronic alcohol use is a global health problem. Statistics show that the global annual per capita consumption of pure alcohol increased from 5.7 liters in 1961 to 9.7 liters in 2002 [102]. Eliasen et al., in their meta-analysis, state that chronic alcohol consumption increases postoperative morbidity, namely general morbidity (RR 1.56, 95% CI 1.31–1.87), overall infections (RR 1.73, 95% CI 1.32–2. 28), wound complications (RR 1.23, 95% CI 1.09–1.40), pulmonary complications (RR 1.80, 95% CI 1.30–2.49), prolonged hospital stay (RR 1.24, 95% CI 1.1–1.31) and intensive care unit admission (RR = 1.29; 95% CI: 1.03–1.61) [103]. In Europe, the prevalence of hazardous drinking among patients undergoing surgery has been reported to be 7–49% for elective surgery and 14–38% for emergency surgery. Preoperative assessment should include evaluating weekly alcohol intake, as reducing consumption to the recommended limits lowers postoperative complications. Brief alcohol interventions provided by healthcare professionals are effective for achieving this goal (significant reduction in postoperative complications) [104].

### 5.5. Psychological Support

Emotional health is as important as physical health, which is why, in recent years, psychological support for patients has garnered significant interest from the researchers. In particular, patients diagnosed with malignancies have been extensively studied. The conclusion is that optimizing psychological status is beneficial for recovery after surgical interventions. Psychological preparation is an important aspect of prehabilitation, with psychological traits, such as mood and attitude, influencing postoperative outcomes. Addressing adverse traits, like depression and anxiety, through preoperative psychological support and interventions, such as education, relaxation and hypnosis, may improve surgical results [105].

Preoperative psychological factors evaluated in studies addressing surgical outcomes include a wide range of mood factors, such as anxiety, depression, psychological stress, hostility, perceived stress and anger. Attitudinal factors are also crucial, encompassing self-efficacy, perceived control, positive expectations, optimism, locus of control and the desire for involvement. Additionally, personality traits play a significant role, with variables like neuroticism, extraversion, self-esteem, motivation, ego strength and feelings of inadequacy impacting surgical outcomes. These factors are interconnected and can significantly influence how patients cope with surgery and their overall recovery process [105,106]. An efficient psychological intervention can enhance motivation and adherence to physical activity and nutrition, acting synergistically in the context of multimodal prehabilitation programs. Furthermore, a hyper-dopaminergic state produced by exercise can be sufficient to overcome aversive events, which can contribute to the development of stress-related psychiatric disorders, such anxiety and depression [107].

In a qualitative systematic review of 48 trials involving 23,037 patients, Ip et al. [108] identified several preoperative factors that predicted acute postoperative pain. Anxiety was found to be one of the four factors consistently linked to postoperative outcomes (95% CI 0.042–0.106, *p* < 0.001), along with age, type of surgery and preoperative pain. Foster et al. [109] conducted a trajectory analysis examining the health and well-being of colorectal cancer patients over a 2-year period, starting with baseline measures taken before surgery and continuing with follow-up assessments at regular intervals. Their findings showed that higher levels of depression before surgery and lower self-efficacy in managing illness were strongly associated with poorer recovery outcomes, even when adjusting for factors such as disease type, treatment and the presence of a stoma. These results emphasize the role of self-efficacy in pain management and support the idea that psychological prehabilitation, focused on enhancing coping skills, may improve recovery.

A multicentric study conducted in 2023 on patients diagnosed with cancer who were about to undergo surgery concluded that prehabilitation and recovery programs can provide significant benefits for participants’ emotional well-being, reducing anxiety, increasing confidence in their ability to cope with treatment and offering an environment where patients can discuss cancer comfortably. Implicit psychological support, provided by exercise specialists, was appreciated by those who did not want explicit psychological support. Additionally, involvement in such a program can reduce anxiety associated with invasive treatments, benefiting postoperative outcomes and contributing to a better quality of life. Prehabilitation programs should include psychological support, but some patients may prefer general emotional support rather than referral to a specialist, due to stigma or associated costs [110].

## 6. Efficacy of Prehabilitation Interventions

To assess the efficacy of multimodal prehabilitation interventions in patients who underwent major gastrointestinal surgery, we reviewed the results of the randomized controlled trials published during the last decade.

### 6.1. Methodology

**Figure 4:**
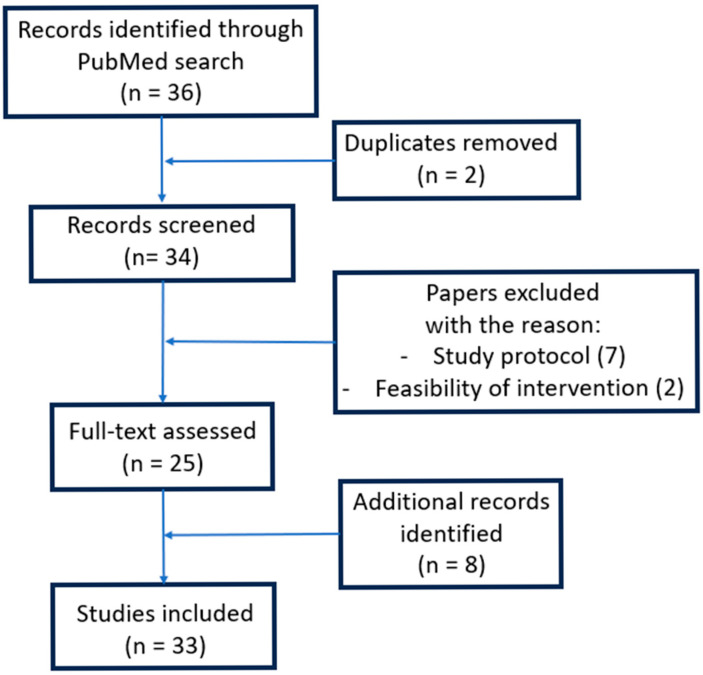
Flowchart of search results.

To retrieve the randomized clinical trials (RCTs) published on this subject in the English literature, we searched the PubMed database using the following terms: ((“major abdominal surgery” [Title/Abstract] OR (“pacreatic” [All Fields] AND “cancer” [Title/Abstract]) OR “gastric cancer” [Title/Abstract] OR “colorectal cancer” [Title/Abstract] OR “hepatobiliopancreatic surgery” [Title/Abstract]) AND “prehabilitation” [Title/Abstract]) AND (randomizedcontrolledtrial [Filter]). The search generated 36 results. The full-text papers were retrieved and evaluated by three authors (N.M.J., C.V.T. and R.V.C). The articles that reported the launch of new trials (including the study protocol only) (7), the feasibility of different interventions (2) and duplicate studies (2) were removed. Two authors (D.G.M. and G.A.P) also evaluated the references of the remaining 25 papers to identify additional RCTs that were not found during the initial search. Thus, another 8 trials were identified and included in the analysis (Figure 4). Out of these 33 RCTs, 21 evaluated patients who underwent colorectal resections (most of them for malignancies), 6 included patients treated by major abdominal surgery, 3 discussed the impact of prehabilitation on patients with (eso)gastric cancers and 2 enrolled patients undergoing HPB surgery. Due to the heterogeneity of the 33 RCTs, we reported the results as a narrative review.

### 6.2. Results

The results of these multimodal interventions are reported considering the type of reported outcome (functional capacity, postoperative complications and cost-effectiveness), according to the type of disease and general condition of the patient (high risk of postoperative complications or frail patients).

Most data derived from the results of patients who were operated on for colorectal (pre)malignant conditions (21 RCTs), or from patients undergoing major abdominal surgery (5 RCTs). The remaining 5 trials dealt with patients operated on for (eso)gastric cancer (3) or hepato-bilio-pancreatic pathology (2).

#### 6.2.1. Functional Capacity

Most studies focused on patients operated on for colorectal cancer and revealed that multimodal prehabilitation improved functional capacity compared to the baseline, although there were few trials that revealed that the difference in functional capacity gain was not significantly different between the experimental and control arm.

Thus, a monocentric RCT found that, at the time of colorectal resection, patients in the prehabilitation group had significantly improved 6MWD (percentage improvement: 131% vs. 107%; *p* < 0.001) and incentive spirometry (113% vs. 100%; *p* < 0.001), compared with the control group [111]. Another RCT observed the effects of multimodal prehabilitation on functional capacity between the time of diagnosis and the moment of surgery, in patients operated on for colorectal cancer. The intervention group achieved a greater increase in the distance covered in the 6MWT (+68.9 m vs. 27.2 m, *p* = 0.01). Furthermore, ergospirometry revealed a significant improvement of functional capacity in the intervention group vs. control (+0.79 METs vs.−0.84 METs, *p* = 0.001) [112].

In a RCT including patients who underwent abdominal surgery, short-term HIIT as part of a multimodal prehabilitation program improved fitness before surgery. Thus, a significant improvement in maximal oxygen consumption was observed in the prehabilitation group vs. control group (VO2peak increase: 2.87 ± 1.94 mL/kg/min vs. 0.15 ± 1.93 mL/kg/min, respectively; 95% CI 1.53–3.93; *p* < 0.001) [72]. Similarly, a secondary analysis of the PREHAB, randomized, clinical trial found that in patients with colorectal cancer, multimodal prehabilitation was associated with improved postoperative functional capacity [113].

Furthermore, a study that pooled patients from 2 other RCTs compared the changes in lean body mass (LBM) and fat mass (FM) in patients with colorectal cancer who underwent trimodal prehabilitation or only rehabilitation. The results showed that prehabilitation positively modulated body composition after operation, with significantly higher absolute and relative LBMs and significantly lower relative and absolute FMs at four and eight weeks postoperatively in the prehabilitation group vs. rehabilitation-only group. The authors concluded that offering a prehabilitation program to colorectal cancer patients awaiting resection is a useful strategy to mitigate the impact of the surgical stress response on lean tissue in the ERAS setting [114]. A randomized trial published in 2018 by Bousquet-Dion et al. showed that the subgroup of patients who were considered to be inactive, irrespective of their age, were more likely to significantly improve their functional exercise capacity if they were in the prehabilitation group (OR 7.07; 95% CI 1.10–45.51) [80]. Furthermore, prehabilitation seems to offer the greatest benefits to frail patients, as shown by Barberan-Garcia et al. in a randomized clinical trial [115]. Their study included 165 frail patients with ASA scores of III-IV who underwent elective, major, abdominal surgery. The patients were divided into two groups, with 62 in the prehabilitation group and 63 in the control group. While in the control group the aerobic capacity did not change, in the prehabilitation group the endurance time (ET) significantly increased (ΔET = 135%; *p* < 0.001) [115]. Chabot et al. conducted a study that followed the effect of supervised multimodal prehabilitation on the functional capacity of prediabetic patients undergoing surgery for non-metastatic colorectal cancer. Two groups of patients were compared, both of whom were ERAS-treated: prediabetic, defined as having glycated hemoglobin between 5.7% and 6.4% at baseline, and the control group—normoglycemic. Each group was then divided into two subgroups: those who had prehabilitation for 4 weeks preoperatively and those who did not. The results show that prehabilitation is the most important factor that can improve functional capacity preoperatively (OR 2.42, 95% CI 1.18, 4.94). Also, the degree of protection of postoperative functional capacity is higher in prediabetic patients enrolled in the prehabilitation program (prehabilitation prediabetics vs. control prediabetics: OR 5.5, 95% CI 1.2–25.8; prehabilitation normoglycemics vs. control normoglycemics: OR 1.5, 95% CI 0.53–4.52) [116].

Prehabilitation also has a significant impact on functional capacity, even in patients with upper gastrointestinal cancers. A RCT recruited 71 patients with esophageal and gastric cancers undergoing neoadjuvant treatment prior to surgery; the patients were allocated to multimodal prehabilitation or standard of care. All patients underwent standardized nutritional care, while exercise prehabilitation (either supervised or home-based interventions) was offered only to the patients in the prehabilitation group. After a median period of prehabilitation of 12.7 weeks (from baseline to pre-surgery), a significant improvement in 6MWT was observed in the prehabilitation group vs. standard of care [+50.7 m, *p* = 0.05; from 522.1 m (104.3) to 582.1 m (108) vs. 497.5 m (106) to 506 m (140)] [117]. Another pilot RCT assessed the impact of multimodal prehabilitation during neoadjuvant therapy on physical capacity in patients operated on for eso-gastric cancer. The authors found that prehabilitation resulted in an attenuated peak VO_2_ decline (*p* = 0.022), less muscle loss (*p* = 0.049) and improved QOL. Significantly more patients who underwent prehabilitation completed neoadjuvant therapy at full dose [18 (75%) vs. 13 (46%); *p* = 0.036] [118].

#### 6.2.2. Length of Hospitalization

Among the 18 RCTs that included data about length of hospital stay, only 4 revealed a marginally significant shorter length of hospitalization in patients who underwent multimodal prehabilitation (Table 1). Two of the studies evaluated patients with colorectal cancer [80,119], one included patients who underwent major abdominal surgery [72,115] and one study enrolled patients with gastric cancer [120].

Although most RCTs did not reveal significant differences in the length of hospitalization between prehabilitation and control groups, with few of them finding a marginal benefit for prehabilitation, a recent meta-analysis of nine studies assessed the impact of nutrition prehabilitation, with or without accompanying exercise, on the short-term outcomes of patients undergoing colorectal surgery. The authors found that receiving any form of prehabilitation significantly reduced hospital stay duration by an average of 2 days compared to the controls (95% CI: −3.5 to −0.9 days) [121].

**Table 1:** Short-term postoperative outcomes achieved my multimodal prehabilitation interventions in the evaluated randomized clinical trials.

Study		Pathology of Interest/Main Inclusion Criteria	Multimodal Prehabilitation	Number of Enrolled Patients		Length of Stay (Days)		ICU Days (Patients Admited in ICU)		Surgical Reintervention		Hospital Readmission		Mortality Rate		Rate of Postoperative Complications		Medical Complications		Surgical Complications	
1	A. Barberan-Garcia et al., Ann. Surg. 2018, 2019 [115]	Elective major abdominal surgery for cancer	Yes	144	73	8 (8)	*p* = 0.078	3 (2)	*p* = 0.046	2 (3%)	*p* = 0.273	2 (3%)	*p* = 0.009	1 (2%)	*p* = 1	19 (31%)	*p* = 0.001	0.2 (0.6) *	*p* < 0.001	0.3 (0.7) *	*p* = 0.119
			No		71	13 (20)		12 (20)		6 (10%)		11 (18%)		1 (2%)		39 (62%)		0.9 (1.2) *		0.5 (0.6) *	
2	A. E. M. Berkel et al., Ann. Surg. 2022 [122]	Elective colorectal resection for cancer	Yes	74	39	8.4 (7.4)	*p* = 0.14			2 (7%)	*p* > 0.99	4 (14%)	*p* > 0.99	1 (4%)	*p* = NS	12 (43%)	*p* = 0.024	8 (29%)	*p* = 0.45	10 (36%)	*p* = 0.14
			No		35	9.1 (7.0)				2 (7%)		5 (17%)		0		21 (72%)		11 (38%)		16 (55%)	
3	C. J. L. Molenaar et al., JAMA Surg. 2023 [123]	Elective colorectal resection for cancer	Yes	269	136	3 (3–5)	*p* = 0.20					4 (3.3%)	*p* = 0.26			39 (28%)	*p* = 0.039	19 (15.4%)	*p* = 0.02	26 (21.1%)	*p* = 0.25
			No		133	3 (3–4)						8 (6.3%)				54 (40%)		35 (27.3%)		35 (27.3%)	
4	A. Fulop et al., Anaesthesia 2021 [111]	Elective colorectal resection for cancer	Yes	149	77	8 (7–10)	*p* = 0.712							1 (1.3%)	*p* = 0.483	17 (22%)	*p* = 0.569				
			No		72	8 (7–9)								2 (2.7%)		16 (22%)					
5	F. Carli et al., JAMA Surgery 2020 [124]	Elective colorectal resection for cancer	Yes	110	55	4 (3–8)	*p* = 0.32					2 (3.6)	*p* = 0.18			25 (45%)	*p* = 0.9	0.56 *	*p* = NS	14 (25%)	*p* = NS
			No		55	5 (3–9)						5 (9.1)				25 (45%)		0.58 *		14 (25%)	
6	C. Gillis et al., Anesthesiology 2014 [1]	Elective colorectal resection for cancer	Yes	77	38	4 (3–5)	*p* = 0.812					6 (15%)	*p* = 0.780			12 (32%)	*p* = 0.277	3 (9%)	*p* = 0.291	9 (24%)	*p* = 0.104
			No		39	4 (3–7)						5 (13%)				17 (44%)		1 (3%)		16 (42%)	
7	S. Gloor et al., Langenbecks Arch. Surg. 2022 [125]	Elective colorectal resection for cancer	Yes	107	54	7 (3–18)	*p* = 0.874	2 (1–3)	*p* = 1	2 (4%)	*p* = 0.495			0		52 (97%)	*p* = 0.169				
			No		53	6 (2–20)		2 (1–2)		0				0		45 (85%)					
8	A. Bausys et al. Br. J. Surg. 2023 [120]	Elective gastric resection for cancer	Yes	122	61	11 (7)	*p* = 0.083					2 (3.4%) ^#^	*p* = 0.163	1 (1.7%) ^#^	*p* = NS	14 (23%) ^#^	*p* = 0.001				
			No		61	13 (9)						7 (11.9%) ^#^		3 (5.1%) ^#^		36 (59%) ^#^					
9	G. Bousquet-Dion et al. Acta Oncologica 2018 [80]	Elective colorectal resection for cancer	Yes	72	41	3 [3–7]	*p* = 0.057					5 (12.2%)	*p* = 0.415	0		14 (38%)	*p* = 0.562				
			No		31	3 [2–4]						2 (6.5%)		0		8 (31%)					
10	F. Ausania et al. Rev Esp Enferm 2019 [126]	Pancreaticoduodenectomy	Yes	40	18	11.4 (7–46)	*p* = 0.449					1 (5.6%)	*p* = 0.673	0		6 (33.3%)	*p* = 0.18				
			No		22	13.2 (7–60)						2 (9.6%)		0		12 (54.5%)					
11	Triguero-Canovas et al. Supportive Care in Cancer 2023 [112]	Elective colorectal resection for cancer	Yes	44	23	5.74 (3.54)	*p* = 0.30									4 (17.4%)	*p* = 0.22				
			No		21	6.67 (3.49)										7 (33.3%)					
12	A. Onerup et al. Ann Surg 2022 [127]	Elective colorectal resection for cancer	Yes	668	317	7 (1–91)	*p* = 0.13			58 (18%)	*p* = 0.04	73 (23%)	*p* = 0.67	3 (1%)	*p* = NS	237 (75%)	*p* = 0.153				
			No		351	6 (1–78)				44 (13%)		76 (22%)		1 (0.3%)		245 (70%)					
13	A. Pesce et al. Surg Endosc 2024 [128]	Elective colorectal resection for cancer	Yes	71	35	5.45 (4.61)	*p* = 0.426	0.4 (2.04)	*p* = 0.246	1 (2.8%)	*p* = 0.572	0	*p* = 0.321	0	*p* = 0.321	7 (20%)	*p* = 0.565				
			No		36	4.8 (1.53)		0.02 (0.16)		2 (5.5%)		1 (2.8%)		1 (2.8%)		8 (22.2%)					
14	A. Hamad BMC Sports Science, Medicine and Rehabilitation 2025 [78]	Gastro-intestinal surgery	Yes	51	17	7.0 (4–7)	*p* = 0.446					2 (12%)	*p* = 0.703			14 (82%)	*p* > 0.999				
			No		34	5.5 (4–8)						6 (18%)				28 (82%)					
15	S. Liang Surgery 2025 [129]	Liver cancers	Yes	205	104	8.51 (3.06)	*p* = 0.765					14 (13.9%)	*p* = 908			94 (91.4%)	*p* = 0.493				
			No		101	8.37 (3.75)						15 (14.4%)				89 (88.1%)					
16	J. Chen BMC Gastroenterology 2024 [130]	Elective gastric resection for cancer	Yes	115	57	8 (6–10)	*p* =0.493	2 (1–3)	*p* = 0.224			3 (5.3%)	*p* = 0.983	1 (1.8%)	*p* = 0.311	6 (10.5%) ^‡^	*p* = 0.033	7 (12.3%)	*p* = 0.025	8 (14%)	*p* = 0.636
			No		58	8 (7–11)		2 (1–4)				3 (5.2%)		0		15 (25.9%) ^‡^		17 (29.3)		10 (17.2)	
17	J. Woodfield Scand J Med Sci Sports 2022 [72]	Major abdominal surgery	Yes	63	28	4 (2.8)	*p* = 0.313									0.64 (±0.95) *	*p* = 0.072				
			No		35	5 (6)										1.16 (±1.11) *					
18	C. Griffiths J Surg Oncol. 2024 [95]	Major abdominal cancer surgery	Yes	63	33											8 (24.2%) ^¶^	*p* = 1				
			No		30											7 (23.3%) ^¶^					
19	López-Rodríguez-Arias Supportive Care in Cancer 2021 [119]	Elective colorectal resection for cancer	Yes	20	10	4.8 (1)	*p* = 0.052									2 (20%)	*p* = 0.16				
			No		10	7.2 (3.2)										5 (50%)					

*—Number of complications per patient; #—at 90 days; n()—number (standard deviation); n( – )—number (interquartile range: 25–75); ‡—CCI > 20 (CCI—comprehensive complication index); ¶—major complications.

#### 6.2.3. Postoperative Complications

Nineteen studies reported the impact of prehabilitation on postoperative complications (Table 1). The impact of prehabilitation on postoperative complications is discussed according to the type of patients’ pathology.

##### Colorectal Cancer

Eleven RCTs assessed the rate of postoperative complications in patients with colorectal resection. Only two of them [122,123] found a significantly lower morbidity rate in patients who underwent prehabilitation. The other 10 studies reported similar morbidity rates between the intervention and control group (Table 1).

Thus, a randomized trial published in 2022 revealed that a 3-week supervised-activity prehabilitation program was associated with significantly lower rates of postoperative complications compared to standard of care (42.9% vs. 72.4%, RR 0.59, 95% CI 0.37–0.96 *p* = 0.024) in high-risk patients (older than 60 years and with a score > 7 metabolic equivalents on the veterans-specific activity questionnaire) who underwent colorectal resections [122]. Furthermore, a recent, well-designed, randomized control trial (PREHAB), which included 251 patients, revealed that a multimodal prehabilitation program was associated with significantly lower rates of postoperative complications and optimized postoperative recovery compared with standard care, in patients operated on for non-metastatic colorectal cancer [123].

##### Gastric/Esophago-Gastric Cancer

Two studies evaluated the impact of multimodal prehabilitation on postoperative complications.

A randomized trial evaluated the impact of multimodal prehabilitation on postoperative outcomes in frail patients with gastric cancer who received up-front surgery. Physical intervention consisted of home-based aerobic exercises and supervised resistance exercises for 3 weeks before surgery. This RCT revealed that significantly more patients in the prehabilitation group achieved an increase in the 6MWD test of at least 20 m (47.4% vs. 27.6%, *p* = 0.028). Although no significant difference was found between the two groups in terms of the 30-day comprehensive complication index (CCI), the incidence of severe complications (CCI > 20) was significantly lower in the prehabilitation group (11.1% vs. 25.9%, *p* = 0.046), mainly due to a significantly lower rate of medical complications (12.3% vs. 29.3%, *p* = 0.025) [130]. On the other hand, Bausys et al. found a lower rate of minor postoperative complications (6.8% versus 42.4%, *p* = 0.001) in patients operated on for gastric cancer after neoadjuvant chemotherapy. However, the rates of severe complications were similar in the two groups (16.9% versus 18.6%, *p* = 0.810) and no significant differences were observed regarding to duration of hospitalization, 90-day mortality and readmission rate during the first 90 days [120].

##### Hepato-Pancreato-Biliary Surgery

The first RCT, which evaluated the impact of prehabilitation on patients undergoing pancreaticoduodenectomy for pancreatic or periampullary carcinoma, recruited 40 patients who were randomized to either multimodal, supervised prehabilitation (18 patients) or standard care (22 patients). After a median duration of prehabilitation of 12.6 days, there were no significant differences between the two groups regarding length of hospital stay (*p* = 0.449), rate of readmissions (*p* = 0.673) and rate of postoperative complications (*p* = 0.18), or major morbidity (*p* = 0.751) or clinically significant pancreatic fistula (0.204) [126]. Only the rate of delayed gastric emptying was significantly lower in the prehabilitation group (*p* = 0.01). This study had some important drawbacks, such as the small sample size and the short duration of the prehabilitation interval (median 12.6 days). Moreover, at least half of the patients in the control group received a sort of prehabilitation (nutritional, unsupervised exercises). All these confounding factors might contribute to the negative results, and the generalizability of these data cannot be taken into account [126]. On the other hand, it seems to be unethical to not offer any type of nutritional support or pancreatic enzyme replacement therapy to patients in the control arm. Similarly, a recent randomized trial, which included 205 patients who underwent liver resection, revealed that a short-term (1-week) exercise-only prehabilitation program was associated with a significant improvement in functional capacity, but postoperative complications and length of hospital stay were not statistically significant between the two groups [129]. The major drawback of this study is the relatively short period of prehabilitation (one week), which may explain its inconclusive results.

Regarding the benefit of prehabilitation in patients undergoing pancreatic or liver resections, although the current RCTs did not show a significant improvement in postoperative outcomes, a recent retrospective analysis with propensity score matching suggested that personalized prehabilitation was associated with fewer cardiopulmonary complications after pancreaticoduodenectomy (9.2% versus 23.3%; *p* = 0.002), although surgical complications and length of stay were not significantly reduced [131].

##### Major Abdominal Surgery

Four RCTs reported postoperative complications in patients receiving major abdominal surgery. One of them revealed a significantly lower rate of postoperative complications in the prehabilitation group [115], another found a marginally significant benefit for patients who received multimodal intervention [72] and the other two studies failed to prove any significant benefits for prehabilitation [78,95]. The last two studies had other primary end-points than postoperative complications, probably being under-powered to evaluate the impact of prehabilitation on postoperative morbidity rates. In the second study, although the postoperative complication rates per patient (0.64 vs. 1.16, *p* = 0.07) and the length of hospital stay (5.5 vs. 7.4 days, *p* = 0.07) decreased, the differences were only marginally significant [72], probably due to the small sample size. Similar to the previous two studies, postoperative complications and length of hospital stay were secondary outcomes. The first RCT was well-designed and its primary end-point was the comparison of postoperative complications between patients who received or did not receive prehabilitation. The study included 165 frail patients with ASA scores of III-IV who underwent elective major abdominal surgery. The patients were randomly assigned to undergo either the endurance-exercise-training-based prehabilitation program (62 patients) or standard of care (63 patients). The number of postoperative complications per patient was significantly lower in the prehabilitation group (0.5 vs. 1.4; *p* = 0.001), mainly due to the significantly lower number of medical complications per patient (0.2 vs. 0.9; *p* < 0.001). Even the rates of postoperative complications and readmissions were significantly reduced in the prehabilitation group (RR 0.5, 95% CI 0.3–0.8, *p* = 0.001; RR 6.4, 95% CI 1.4–30, *p* = 0.009) [132].

##### Elderly/Frail Patients

Because the highest rates of postoperative complications are associated with advanced age, frailty and reduced physical activity, studies concerning the benefits of prehabilitation in such patients are tremendously important. For example, in a study conducted by Busquet-Dion et al., the subgroup of patients who were considered to be inactive, irrespective of their age, were more likely to significantly improve their functional exercise capacity (OR 7.07; 95% CI 1.10–45.51) and accelerate their recovery after surgery if they were in the prehabilitation group [80].

As mentioned above, Barberan-Garcia et al. showed that frail patients who underwent major abdominal surgery for digestive cancers significantly benefited in terms of postoperative complications from multimodal prehabilitation interventions [115]. Similarly, another randomized trial published in 2022 revealed that exercise prehabilitation was associated with significantly lower rates of postoperative complications (RR 0.59, 95% CI 0.37–0.96 *p* = 0.024) in high-risk patients who underwent colorectal resections [122].

Furthermore, a RCT compared the short-term outcomes (the primary end-point was the comprehensive complication index—CCI) achieved by multimodal prehabilitation vs. multimodal rehabilitation in frail patients operated on for colorectal cancer. Exercise intervention consisted of a 4-week program with 1-day supervision. No differences between groups were observed, either regarding CCI (*p* = 0.45) or overall complications, severe complications, emergency department visits and readmissions [124]. However, a secondary analysis of the above-mentioned RCT followed patients who received prehabilitation and divided them into 2 subgroups: those who could walk a minimum of 400 m in a maximum 6 min and those who could not, after 4 weeks of prehabilitation. Out of 55 enrolled patients, 8 failed to walk for 6 min and 28 were unable to reach and/or exceed the 400 m threshold in 6 min. The patients who failed to reach the 400 m threshold in 6 min were 6.2-times more likely to develop a postoperative complication (OR 6.2, 95% CI: 1.1 to 36.1; *p* = 0.041) [133]. This observation emphasizes the importance of a personalized efficient prehabilitation program aiming to help frail patients to achieve specific functional-capacity targets before surgery.

Although the risk of developing postoperative complications is significantly lower in fit patients, it seems that also these patients can benefit from multimodal prehabilitation interventions. In a small study, Bojesen et al. enrolled 36 patients with WHO performance status I or II who underwent elective colorectal cancer surgery. The intervention group received a multimodal prehabilitation program, and postoperative effects were quantified using a validated questionnaire with a score range between 0 and 150 called Quality of Recovery-15. The results showed that the intervention group scored 21.9 (95% CI 4.5–39.3) more points in the first 3 postoperative days than the control group on the Quality of Recovery-15 questionnaire [134].

#### 6.2.4. Cost-Effectiveness

To assess the impact of nutritional status on hospitalization costs, Guerra et al. [38] conducted a prospective study in a university hospital on a group of 637 patients. The nutritional status of the patients was evaluated within the first 72 h of hospitalization and the individual cost of hospitalization was determined using the diagnosis-related group system. Thus, they showed that the risk of undernutrition according to Nutritional Risk Screening (NRS-2002) and the high risk of undernutrition according to the Malnutrition Universal Screening Tool (MUST) increased patient costs by 21.1% [95% CI = 9.0–33.2%] and 28.8% [95% CI = 13.7–39.9%], respectively. Also, severe undernutrition according to the clinical characteristics recommended by the Academy of Nutrition and Dietetics (AND) and American Society for Parenteral and Enteral Nutrition (ASPEN) and the Patient Generated Subjective Global Assessment (PG-SGA) was associated with higher hospitalization costs—19.4% [95% CI = 7.3–31.5%] and 27.5% [95% CI = 14.0–41.1%], respectively. Thus, the cost of a patient at nutritional risk or who is undernourished is between EUR 416 (95% CI = EUR 156–675) and EUR 617 (95% CI = EUR 293–855) higher than the average of the same case type.

Regarding the cost-effectiveness of prehabilitation, important information has been offered by a cost analysis based on the secondary results of a RCT, which compared the results of prehabilitation vs. standard of care in elderly, frail patients who underwent major abdominal surgery [132]. This analysis revealed that the costs of an endurance-exercise-training-based prehabilitation program was 389 EUR/patient, which seems to be lower than the above-mentioned supplementary cost of a patient at nutritional risk or who is undernourished. Furthermore, Barberan-Garcia et al. revealed that the rate of 30-day hospital readmission was significantly lower in the prehabilitation group than in the standard of care group, resulting in a significant increase in the average cost savings of prehabilitation by including healthcare use at the 30-day follow-up (compared with considering only the initial hospitalization) (EUR 333 [745] vs. EUR 812 [894]; *p* < 0.001) [132]. These results support the efficacy and cost-effectiveness of a prehabilitation program [132].

### 6.3. Discussion

The lack of uniformity in terms of the efficacy of prehabilitation can be explained by the broad spectrum of physical interventions applied, different types of pathological conditions of the enrolled patients and different interventions performed in the control group.

The types of physical exercises included in the prehabilitation program, as well as the supervision of the participant, seem to be of paramount importance to achieve a satisfactory improvement of functional capacity and an improved recovery after operation. Thus, the PHYSSURG-C trial, which assessed the impact of a home-based physical prehabilitation program consisting of 30 min of daily aerobic activity plus inspiratory muscle training in patients who underwent elective colorectal resection, revealed that there were no significant differences between the groups in any of the evaluated parameters [127]. This observation suggests that resistance exercises might represent the cornerstone method that should be used in physical prehabilitation to improve functional capacity and postoperative recovery. Furthermore, a pooled analysis of the patients with colorectal cancer enrolled in two randomized clinical trials at the same center revealed that a prehabilitation program including supervised exercise training significantly enhanced functional capacity and muscle strength when compared to the prehabilitation interventions without supervision (*p* < 0.01). The patients receiving exercise supervision had more than two-times-higher chances to return to baseline after surgery. The odds ratio to return to the baseline characteristics of the supervised prehabilitation group vs. non-supervised-rehabilitation-only group was 7.71 at 4 weeks (95% CI 2.06–28.82) and 8.62 at 8 weeks (95% CI 2.44–30.49) [135]. Anyway, there are situations when prehabilitation programs cannot be carried out in health facilities under the close supervision of specialized staff, and a good example is the global COVID-19 pandemic. In the lockdown period from 13 March to 21 June 2020, López-Rodríguez-Arias et al. conducted a study examining the effects of multimodal prehabilitation performed at home in patients undergoing elective surgery for colorectal cancer. The results showed that even in this situation, there was a marginally significant reduction in hospital stay (4.8 vs. 7.2 days, *p* = 0.052) in the home prehabilitation group, without a statistically significant reduction in the postoperative complication rate (20% vs. 50%, *p* = 0.16) compared to the control group. Moreover, at 45 days postoperatively, the decrease in lean mass was lower in the intervention group than in the control group (1.7% vs. 7.1%, *p* = 0.17). This parameter almost equalized at 90 days postoperatively [119].

Although most studies and meta-analyses revealed that exercise prehabilitation was associated with improved functional capacity, the data on postoperative outcomes are still conflicting [136]. These conflicting results might be due to the different populations enrolled in these trials and to the different types of exercise prehabilitation interventions that were employed. For example, a recent RCT aimed to assess the efficacy of a 4-week, multimodal, prehabilitation intervention program in terms of functional reserve, length of stay and rate of postoperative complications in patients with colorectal cancer and ASA scores of 1–3. An interim analysis of 71 patients revealed that, although the prehabilitation group achieved a significant increase in the mean 6MWT distance preoperatively (96 m; 523 ± 24.6 vs. 427 ± 25.3, *p* = 0.01), no statistically significant differences were observed between the two groups regarding the rates of postoperative complications and length of hospitalization [128]. This may be explained by the fact that most patients were fit for surgery, as their ASA score was 1–3.

Furthermore, a RCT (PHYSSURG-C) that assessed the impact of a home-based physical prehabilitation program consisting of 30 min of daily aerobic activity plus inspiratory muscle training in patients who underwent elective colorectal resection revealed that there were no significant differences between the groups for the comprehensive complication index (CCI) at 30 or 90 days postoperatively, length of hospital stays or readmission rates at 90 days [127]. An updated analysis after a 12-month follow-up also did not find any effect from the intervention on self-reported physical recovery (OR 0.91, *p* = 0.60), risk of reoperation (OR 0.97, *p* = 0.91) or readmission (OR 0.88, *p* = 0.58) [137]. The lack of significant difference regarding the postoperative complications could be explained by the fact that resistance exercises were not employed in the physical prehabilitation program.

In another study that did not find any difference in the rates of postoperative morbidity between the prehabilitation arm and control arm, the control group included patients who underwent postoperative rehabilitation. Thus, multimodal rehabilitation interventions could enhance recovery in the control arm, explaining the lack of difference in terms of postoperative complications between the two groups [124]. However, considering that the rehabilitation program starts after discharge, its impact on postoperative outcomes could influence the readmission rate, the rate of complications developed after discharge and the total length of total stay, but not the rate of postoperative complications developed during the initial hospital stay.

In order to encourage patients to achieve the exercise targets and to increase their adherence to the prehabilitation program, a modality for the evaluation of self-achievements can be useful. Thus, a feasible option for monitoring physical activity and vital parameters can be the use of smart watches, as shown by Waller et al. They supervised patients enrolled in the prehabilitation program by using smartwatches. The results showed that the intervention group displayed an increase in the distance covered in the 6MWT compared to the control group (+85.6 m, 95% CI, 18.06 to 153.21 vs. +13.23 m, 95% CI 6.78 to 33.23; *p* = 0.014). Importantly, this improved physical fitness translated in a shorter duration of postoperative hospitalization with about 2 days in the prehabilitation group vs. the non-prehabilitation group [138].

Also, nutritional intervention is of tremendous importance for improving postoperative recovery and reducing long-term complications associated with muscle wasting and sarcopenia. Thus, Zhang et al. [139] performed a meta-analysis of 56 studies, including 6370 patients who had surgery for gastrointestinal cancers, and demonstrated that perioperative nutritional supplementation significantly reduced postoperative complications (RR 0.74, 95% CI 0.69–0.80), both infectious (RR 0.71, 95% CI 0.64–0.79) and non-infectious (RR 0.79, 95% CI 0.71–0.87). They also found that length of hospitalization was significantly shorter in patients who received perioperative nutritional supplementation (95% CI −1.83 to −1.32).

Because the reported impact of prehabilitation on postoperative outcomes is not concordant between different studies, Moran and colleagues [140] performed a meta-analysis of the studies, which evaluated the impact of exercise prehabilitation on patients undergoing intra-abdominal surgery. They found that this intervention significantly reduced the incidence of postoperative complications (OR 0.59, 95% CI 0.38–0.91; *p* = 0.03), especially of pulmonary complications (OR 0.27, 95% CI: 0.13–0.57; *p* = 0.0005).

Regarding the impact of multimodal prehabilitation on psychological outcomes, Taha et al. conducted a study examining the effects of the prehabilitation program on preoperative depression and anxiety in patients undergoing colorectal surgery. The degrees of depression and anxiety were quantified by the Hospital Anxiety and Depression Scale (HADS). The results showed that there was no significant improvement in anxiety and depression due to the prehabilitation program [141]. However, a RCT conducted by S. Atoui et al. revealed that in patients with colorectal cancer, prehabilitation intervention was associated with a slightly positive change in the perceived sleep quality before operation (d = 0.11, 95% CI −2.1 to −0.1, *p*  =  0.048). Furthermore, in patients with high anxiety, prehabilitation led to a significant improvement in the rate of change in sleep duration over time (compared to the standard of care), with a difference of 110 min between the baseline and 8 weeks after surgery (d = 0.51, 95% CI: 92.3 to 127.7, *p* = 0.02) [142].

Moreover, a study that evaluated the patients’ perspective of being recruited in a RCT of weight loss intervention before colorectal surgery found that patients were motivated to take part by the prospect of improved surgical outcomes. But, the patients who were randomized to usual care were disappointed, mainly due to the clinical teams’ overemphasis on the benefits of losing weight. The strong preference to be allocated to the intervention suggests that balanced communication is crucial to minimize disappointment from randomization to usual care and differential dropout from the trial [143].

Although there is contradictory evidence regarding the efficacy of prehabilitation in general, expert opinion, based on the current evidence, recommends prehabilitation in elderly high-risk or frail patients who will undergo colorectal and upper gastro-intestinal surgery [144]. Furthermore, supervised resistance exercises seem to be of tremendous importance to achieve lower morbidity rates and shorter length of hospital stay.

## 7. Limitations and Future Directions

Most of the RCTs that reported conflicting results regarding the postoperative complications have major drawbacks and limitations. The majority of them aimed to evaluate the impact of prehabilitation programs on functional capacity (cardio-respiratory fitness), explaining the positive results regarding this outcome. Consequently, most of them were not designed to evaluate the impact on postoperative complications, due to the relatively small number of patients. Thus, the majority was under-powered to identify a significant difference in rates of postoperative complications. Only seven RCTs aimed to assess as a primary outcome the impact of multimodal prehabilitation on postoperative complications [53,115,120,124,129,130,145], and another one has as a primary end-point the self-assessed physical recovery 4 weeks postoperatively [137]. Five of these studies were able to identify a significant difference in postoperative complications between prehabilitated patients and the standard of care group. The other three trials did not achieve the primary end-point. In one of them, the participants in the prehabilitation arm received only one week of unsupervised exercise training [129] and in the study of Onerup et al., more than 75% of patients had an ASA score of 1 or 2 and the training consisted of 2 weeks of home-based aerobic exercises [137]. The short-term exercise activity and the unsupervised training may represent important flaws contributing to the sub-optimal postoperative outcomes in the prehabilitation group. Furthermore, only a limited percentage of patients in the control group were at a high risk of postoperative complications. All these confounding factors may contribute to the contradictory findings reported by these RCTs.

These observations highlight the need to perform well-designed, randomized, controlled trials able to assess if multimodal prehabilitation significantly improves the length of hospital stay and the rate of postoperative complications in patients who undergo major abdominal surgery. In future well-designed trials, the targeted study population should be represented mainly by high-risk (elderly, frail) patients who seem to derive the greatest benefit from prehabilitation, and the predefined sample size should be correlated with the specific primary end-points (e.g., total number of complications, surgical complications, medical complications and major complications according to the Clavien-Dindo classification or the CCI index). Exercise should be based on supervised strength training (3 days weekly) and home-based aerobic exercises (4 days weekly), for 3–4 weeks before surgery. Furthermore, the current evidence suggests that exercise prehabilitation alone might not be sufficient to improve postoperative recovery and it should be combined with nutritional and psychological interventions to increase the effectiveness of these approaches.

A balanced presentation by the medical team of the benefits and risks associated with the interventions from each arm of study is also important, to decrease the drop-out rate, especially in the control arm.

## 8. Conclusions

Multidisciplinarity is crucial in prehabilitation, as collaboration among specialists is essential. In addition to the anesthesiologist and the surgeon, who are directly involved in the surgical procedure, this stage also includes nutritionists, psychiatrists, psychologists, physiotherapists, physical therapists and fitness instructors. The key benefit of prehabilitation is the improved physiological reserve. Although it was hypothesized that the enhanced functional reserve would be associated with improved recovery, the current evidence regarding the impact of prehabilitation on the short-term postoperative results is still contradictory, when we consider all the patients who receive surgery, irrespective their initial fitness. Even if few studies did not show any improvement in the postoperative outcomes, some recent, well-designed, randomized clinical trials revealed that a multimodal prehabilitation program consisting of personalized physical activity and nutritional and psychological support was associated with shorter hospital stay and reduced postoperative complications (especially respiratory and cardiovascular) in patients operated on for digestive malignancies, such as colorectal and gastric cancers. To date, frail patients who underwent supervised resistance exercises and home-based aerobic training seem to derive the greatest benefit from multimodal prehabilitation, in the setting of major abdominal surgery.

## Data Availability

No new data were created or analyzed in this study. Data sharing is not applicable to this article.

## Abbreviations

The following abbreviations are used in this manuscript:

CPET	Cardio-pulmonary exercise test
RCT	Randomized clinical trial
6MWT	6 min walking test
ERAS	Enhanced recovery after surgery
